# Supplementary material for: Should Microhematuria Be Incorporated into the 2023 Duke-International Society for Cardiovascular Infectious Diseases Minor Immunological Criteria?
Source: Antibiotics (Basel). 2025 Jul 7;14(7):687. doi: 10.3390/antibiotics14070687 (PMC12291632; doi:10.3390/antibiotics14070687)
Supplement: Supplementary file 1 [file antibiotics-14-00687-s001.zip › antibiotics-3722591-supplementary.pdf]

Supplementary Table S1. Multivariable analysis of predictors of microhematuria upon presentation among episodes with suspected infective endocarditis

	p	**aOR (95% CI)**
Female sex	0.007	1.60 (1.14–2.26)
Age >60 years	0.162	1.27 (0.91–1.77)
Chronic kidney disease (eGFR<60 ml/min/1,73 m^2)	0.758	1.06 (0.74–1.52)
Diabetes mellitus	0.326	1.19 (0.84–1.70)
S. aureus bacteremia	0.469	1.13 (0.82–156)
Enterococcal bacteremia	0.001	2.79 (1.55–5.04)
Sepsis or septic shock	0.002	1.67 (1.20–2.31)
Non-cerebral embolic events	0.002	2.08 (1.32–3.26)
Acute kidney injury upon presentation	0.022	1.47 (1.06–2.05)
Infective endocarditis	0.622	0.92 (0.65–1.30)
Bone and joint infection[b]	0.001	2.09 (1.35–3.24)

aOR: adjusted odds ratio; eGFR: estimated Glomerular Filtration Rate; CI: confidence interval

[a]as described by the 2023 International Society of Cardiovascular Infectious Diseases Duke criteria

[b]excluding chronic osteitis

Supplementary Table S2. Comparison of episodes with infective endocarditis with and without microhematuria upon presentation

	**Without microhematuria (n = 103)**	**With microhematuria (n = 160)**	***p***
Demographics			
Male sex	82 (80)	126 (79)	1.000
Age (years)	67 (50–75)	66 (50–74)	0.941
Age >60 years	64 (62)	99 (62)	1.000
Comorbidities			
Diabetes mellitus	18 (18)	34 (21)	0.527
Obesity (body mass index ≥30 kg/m^2)	19 (18)	31 (19)	0.874
Chronic kidney disease (eGFR<60 ml/min/1,73 m^2)	15 (15)	31 (19)	0.406
Malignancy (solid organ or haematologic)	13 (13)	21 (13)	1.000
Chronic obstructive pulmonary disease	9 (9)	20 (13)	0.422
Cirrhosis	10 (10)	12 (8)	0.649
Congestive heart failure	11 (11)	18 (11)	1.000
Isolated pathogen			
S. aureus	33 (32)	78 (49)	0.010
Coagulase-negative staphylococci	6 (6)	8 (5)	0.788
Streptococcus spp	40 (39)	31 (19)	0.001
Enterococcus spp	8 (8)	26 (16)	0.059
Other Gram-positive	55(5)	4 (3)	0.320
HACEK	0 (0)	3 (2)	0.283
Gram-negative other than HACEK	5 (5)	11 (7)	0.603
Fungi	0 (0)	5 (3)	0.160
Intracellular pathogens	1 (1)	0 (0)	0.392
No identification	8 (8)	2 (1)	0.016
Manifestations			
Fever (temperature >38°C)	87 (85)	145 (91)	0.170
Sepsis or septic shock	39 (38)	86 (54)	0.016
Embolic events upon presentation[a]	39 (38)	95 (59)	0.001
Cerebral embolic events	20 (19)	46 (29)	0.109
Non-cerebral embolic events	27 (26)	75 (47)	0.001
Immunological phenomena[a]	8 (8)	20 (13)	0.306
Glomerulonephritis[a]	0 (0)	9 (6)	0.013
Bone and joint infections[b]	14 (14)	36 (23)	0.078
Septic arthritis	6 (6)	20 (13)	0.092
Vertebral and non-vertebral osteomyelitis	6 (6)	17 (11)	0.263

Osteoarticular implant-associated infection	1 (1)	2 (1)	1.000
Renal function upon presentation			
Creatinine (umol/L)	88 (69–118)	133 (95–184)	<0.001
Acute kidney injury	26 (25)	84 (53)	<0.001
Stage I	20 (77)	44 (52)	0.052
Stage II	5 (19)	15 (18)	
Stage III	1 (4)	25 (30)	
Site of infection			
Aortic valve	46 (45)	84 (53)	0.246
Mitral valve	41 (40)	64 (40)	1.000
Tricuspid valve	12 (12)	18 (11)	1.000
Pulmonary valve	4 (4)	1 (0.6)	0.079
CIED-lead	8 (8)	23 (14)	0.120
Type of valve			
Native	61 (59)	109 (68)	0.148
Prosthetic	37 (36)	38 (24)	0.037
Intracardiac lesions			
Vegetation	60 (58)	101 (63)	0.440
Vegetation ≥10 mm	36 (35)	71 (44)	0.157
Abscess	13 (13)	25 (16)	0.591
Other lesions[c]	13 (13)	21 (13)	1.000

Data are depicted as number (%) or median (interquartile range)

[a]as described by the 2023 International Society of Cardiovascular Infectious Diseases Duke criteria

[b]excluding chronic osteitis

[c]perforation, dehiscence of prosthetic valve, fistula, aneurysm, pseudoaneurysm

CIED: cardiac implantable electronic devices; eGFR: estimated Glomerular Filtration Rate; HACEK: *Haemophilus* spp, *Aggregatibacter* spp, *Cardiobacterium hominis*, *Eikenella corrodens*, *Kingella kingae*

Supplementary Table S3. Multivariable analysis of predictors of microhematuria upon presentation among infective endocarditis episodes

	p	**aOR (95% CI)**
Chronic kidney disease (eGFR<60 ml/min/1,73 m^2)	0.248	1.56 (0.73–3.34)
Diabetes mellitus	0.794	0.91 (0.44–1.87)
S. aureus endocarditis	0.736	1.12 (0.58–2.17)
Enterococcal endocarditis	0.052	2.53 (0.99–6.42)
Sepsis or septic shock	0.289	1.42 (0.74–2.69)
Non-cerebral embolic events[a]	0.004	2.40 (1.32–4.36)
Acute kidney injury upon presentation	0.001	2.83 (1.50–5.34)
Endocarditis non-related to prosthetic valve	0.018	2.14 (1.14–4.04)

aOR: adjusted odds ratio; eGFR: estimated Glomerular Filtration Rate; CI: confidence interval

[a]as described by the 2023 International Society of Cardiovascular Infectious Diseases Duke criteria
